# Percutaneous Transforaminal Endoscopic Decompression for Lumbar Lateral Recess Stenosis

**DOI:** 10.3389/fsurg.2021.631419

**Published:** 2021-08-05

**Authors:** Hui-gen Lu, Xue-kang Pan, Min-jie Hu, Jian-qiao Zhang, Jian-ming Sheng, Bao Chen, Xiao Zhou, Yefeng Yu, Xu-qi Hu

**Affiliations:** Department of Orthopaedics, The Second Affiliated Hospital of Jiaxing University, Jiaxing, China

**Keywords:** spinal endoscopy, oral decompression, lateral approach, lumbar spinal stenosis, intervertebral disc herniation

## Abstract

**Objective:** The aim of this study was to evaluate the treatment efficacy of lateral spinal stenosis through the decompression of the nerve root under a multiple planar endoscope.

**Methods:** From January 2017 to March 2019, 52 patients with lumbar spinal stenosis or lumbar spinal stenosis combined with intervertebral disc herniation had been treated via transforaminal approach spinal endoscopy. Our study retrospectively analyzed the treatment outcome. All patients experienced complications with different degrees of facet joint hyperplasia and ligamentum flavum hyperplasia and hypertrophy. Some patients suffered disc herniation. All patients were treated with percutaneous transforaminal approach multiple planar endoscopic decompression. The visual analog scale (VAS) and the Oswestry Disability Index (ODI) were compared before and after the operation, as were the horizontal foramen areas of the medial margins of the upper and lower pedicles of the vertebral arch. The treatment effectiveness was evaluated.

**Results:** VAS and ODI scores were significantly improved at postoperative 3 days, 3 months, 6 months, and the last follow-up (*P* < 0.05). The area of the intervertebral foramen was 422.5 ± 159.2 mm^2^ preoperatively and 890.8 ± 367.7 mm^2^ postoperatively, the difference was statistically significant (*P* < 0.05).

**Conclusion:** Percutaneous transforaminal approach multiple planar endoscopic decompression could achieve an accurate and effective decompression of the lumbar lateral spinal canal. This procedure has good short-term effects, and is especially suitable for elderly patients.

## Introduction

Lumbar spinal stenosis is a common degenerative disease. As early as 1954, Verbiest divide it into three types according to the sites: lateral spinal stenosis, central spinal stenosis, and foramen stenosis. The incidence of the first type was the highest (1). Since Kambin and Gelmanns (2) and Hijikata (3) designed the posterolateral approach for spinal endoscopy, percutaneous endoscopic lumbar discectomy has been popularized and standardized. Meanwhile, with the progress of endoscopic technology, including the effective use of endoscopic tools such as motor grinding drills and circular saws, the indications of spinal endoscopy are also expanding from soft disc herniation to rigid spinal stenosis (4). However, endoscopic lumbar discectomy continues to entail difficulties in terms of the decompression of the hypertrophic articular process and the ligamentum flavum (5). In fact, the endoscopic treatment of lateral spinal stenosis requires a ventral or dorsal decompression of the nerve root canal, which demands a three-dimensional view of the stenosis. This study describes the methods of three-dimensional decompression and the attendant short-term outcomes.

## Materials and Methods

### Overview

From January 2017 to March 2019, 52 patients with lumbar spinal stenosis or lumbar spinal stenosis combined with intervertebral disc herniation, who had been treated with percutaneous transforaminal approach spinal endoscopy and had complete clinical data were enrolled in this study. The patients should meet the following inclusion criteria: single level lateral spinal stenosis with or without lumbar disc herniation, patients experienced typical nerve root symptoms or neurogenic intermittent claudication and received non-surgical treatment before the operation. Meanwhile, the patients were excluded if they did not meet the inclusion criteria: Patients experienced some instability of the lumbar spine segment to be operated on (according to the preoperative dynamic film of the lumbar spine), had a history of lumbar surgery or suffered from some form of mental illness. The patients underwent preoperative routine X-ray imaging of the lumbar spine in the anteroposterior and lateral position and dynamic X-ray imaging in the hyperextension and hyperflexion position to judge the stability of the lumbar spine before they underwent thin-layer computed tomography (CT, 1.5 mm) of the lumbar spine and were diagnosed with lumbar lateral spinal stenosis. At this point, an imaging measurement was performed (6). Meanwhile, the patients underwent lumbar magnetic resonance imaging (MRI) to observe the transverse and sagittal morphology of the spinal canal and the disc herniation. The patients with spinal stenosis without any rupture of the annulus fibrosus and the posterior longitudinal ligament underwent decompression alone, while those with disc herniation and dural sac compression were treated via discectomy in addition to bony decompression. In addition, a detailed physical examination was carried out to check the movement, sensation, and reflex of the affected limb, while the symptomatic segments were determined, and the responsible nerve root was identified via the imaging results. Since it is difficult to determine the responsible nerve root in elderly patients due to the multiple space degenerative imaging changes, selective nerve root block (SNRB) was performed to overcome this issue.

### Surgical Procedures

**Figure 1:**
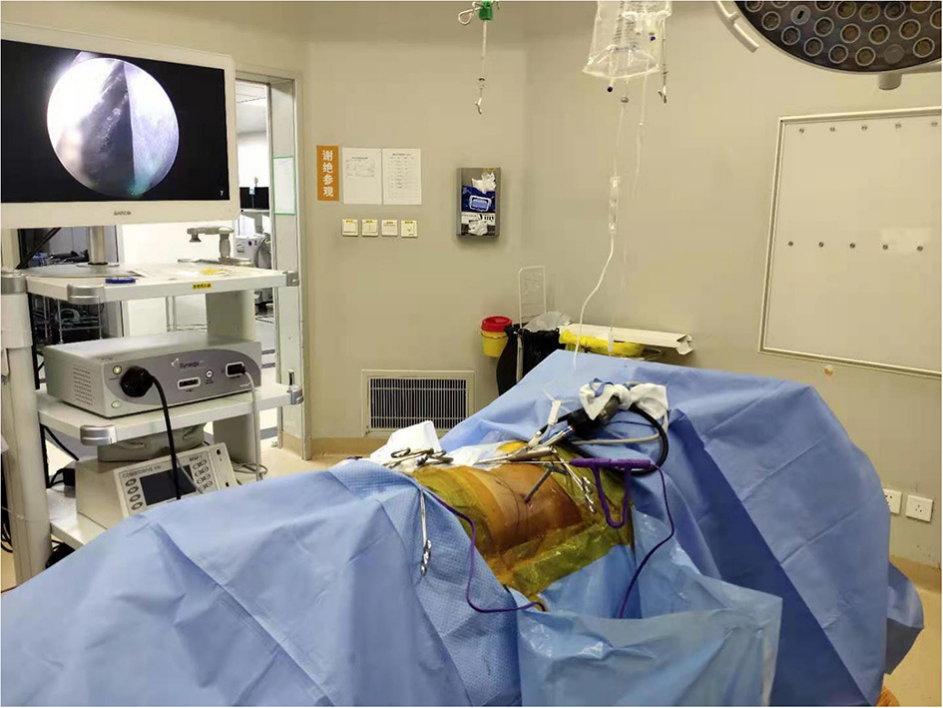
Patient's intraoperative position.

**Figure 2:**
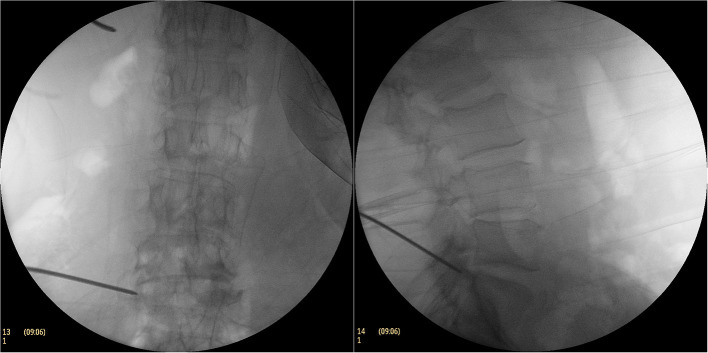
During the operation, Kirschner needle puncture C-arm fluoroscopy. The anchor point of the Kirschner needle was close to the ventral edge of the superior articular process on the lateral fluoroscopic image and the tip of the Kirschner needle was located on the lateral side of the superior articular process on the AP fluoroscopic image.

Taking the L5/S1 space as an example, the patient was placed in a lateral position (Figure 1) and following the administration of local infiltration anesthesia with 1% lidocaine at the point of puncture. Then the puncture needle was inserted to the upper articular process of the S1 vertebral body under an anteroposterior and lateral X-ray fluoroscope, while 1% lidocaine was used for local infiltration anesthesia around the articular process. The puncture core was taken out and a 1.5-mm-diametered Kirschner needle was used as the puncture guide needle, with the tip anchored to the superior articular process. The anchor point was close to the ventral edge of the superior articular process on the lateral fluoroscopic images and the tip of the Kirschner needle was located on the lateral side of the superior articular process, while along the line of the Kirschner wire, it reached the inner upper edge of the pedicle and formed an angle of ~25° with the upper endplate of the vertebral body (Figure 2).

**Figure 3:**
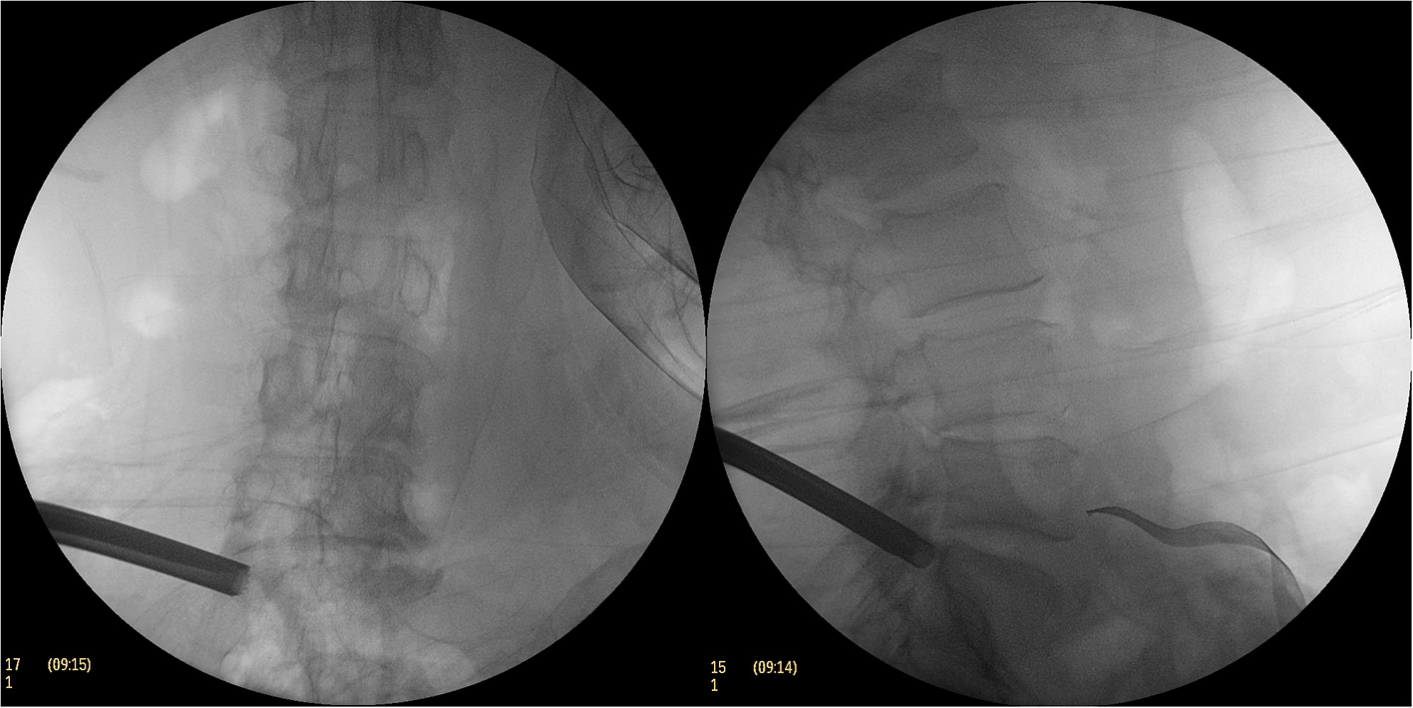
The C-arm fluoroscopic picture of the excised part of the superior articular process with a circular saw.

**Figure 4:**
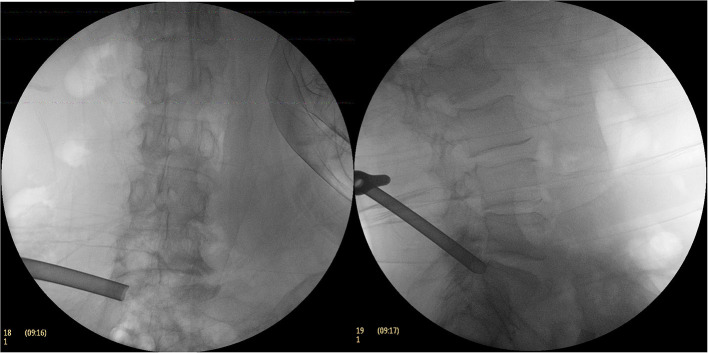
During the operation, working cannula was placed in C-arm fluoroscopy.

An approximate 8-mm-long incision was made and the catheter was expanded step by step along the Kirschner wire before a soft tissue protective cannula was inserted and a circular saw was inserted into the protective cannula to grind the upper articular process of the S1 and the upper medial edge of the partial pedicle (Figure 3). The circular saw was then taken out and a 6.9-mm-diametered working casing (ASAP) was inserted (Figure 4). A transforaminal endoscope was then placed through the working casing and the residual bone fragments were removed with various types of grasping forceps before the ventral side of the superior articular process and the upper edge of the pedicle were exposed. Then, part of the ligamentum flavum was excised to expose the dorsal side of the nerve root, the tissue along the dorsal midline of the nerve root was opened, and the ligamentum flavum attached to the upper edge of the upper lamina was incised. Following the resection of the ligamentum flavum, it was exposed to the intervertebral disc level along the dorsal side of the nerve root.

When exposed to the head side, the hypertrophic superior articular process is often covered by osteotome, and in this case, the hypertrophic superior articular processes are removed using an endoscopic bone chisel or via motor removal; it was exposed to the intervertebral space toward the head end and the dorsal ligamentum flavum and the joint capsule were excised and decompressed. At the level of the intervertebral disc, the working casing of the transforaminal endoscope was adjusted ventrally and the ventral side of the nerve root was explored to check the integrity of the posterior longitudinal ligament.

**Figure 5:**
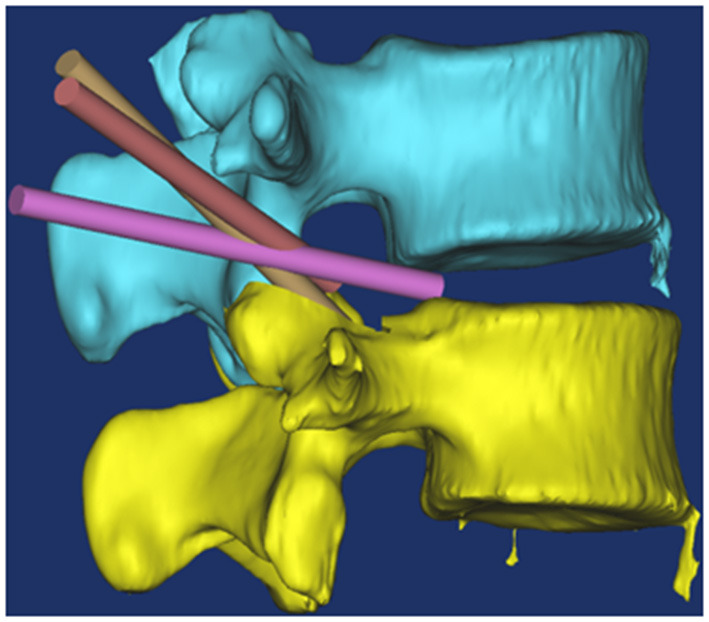
Schematic diagram of endoscopic multi-plane nerve root canal decompression technique. The yellow channel position displayed that directly decompresses superior segment of the mid zone of the lateral lumbar spinal canal; Brown channel position displayed that the working casing was adjusted, the posterior longitudinal ligament and annulus fibrosus were incised; Purple channel displayed that the herniated disc tissue was removed.

For the patients with simple lateral spinal stenosis, the operation was finished once the above procedures had been completed, while for those with disc herniation, further procedures were required. Here, following the dorsal decompression of the nerve root, at the level of the intervertebral disc, the working casing was adjusted ventrally, the posterior longitudinal ligament and annulus fibrosus were incised, the herniated disc tissue was removed. Finally, the ventral and dorsal sides of the nerve root were then checked to determine the presence of any compression. Therefore, the entrance zone and superior segment of the mid zone of the lateral lumbar spinal canal was decompressed. We called it multiple planar endoscopic decompression technology (Figure 5). No steroid drugs were injected into the spinal canal. All the patients underwent a straight leg raising test after the operation, with all the results negative. Endoscopic monitoring indicated no compression on the ventral or dorsal side of the nerve root.

In this study, the operation involved decompressing the bony structure first and then the non-bony structure. This Working casing inserting technology directly decompresses entrance zone and superior segment of the mid zone of the lateral lumbar spinal canal. The remnant bone fragments and thickened ligamentum flavum on the dorsal side of the nerve root were removed with the use of an endoscope. The cannula was swayed to the head side along the articular process, and, with the osteotome under an endoscope, the head part of the superior articular process was excised from the outside to the inside to decompress the superior articular process and the ligamentum flavum on the dorsal side of the entrance area of the lateral vertebral canal. When the swing cannula was decompressed to the level of the intervertebral disc, the cannula was shifted ventrally to the intervertebral disc area to explore the ventral part of the nerve root.

In the patients with simple lateral spinal stenosis, the integrity of the ventral posterior longitudinal ligament of the nerve root was explored, with the operation halted when no compression was found. For the patients with intervertebral disc herniation, the protruded intervertebral disc on the ventral nerve root was removed.

### Postoperative Measures

After the operation, the patients were placed in the supine position and a straight leg raising test was performed. A postoperative X-ray and CT examinations were carried out within 3 days of the operation. Since the structure of the upper and lower pedicles of the vertebral arch is constant, the area measurement of the medial margin of the intervertebral foramen is relatively stable. Therefore, the area between the upper and lower pedicle of the vertebral arch and the medial margin of the pedicle was measured as the intervertebral foramen area. The images were reconstructed via PACS medicine image information system with a CT soft-tissue window before and 3 days after the operation. Then the intervertebral foramen area was calculated by PACS medicine image information system. The VAS and ODI scores were measured at 3 days, 3 months, 6 months, and a final follow-up date. The before and after VAS and ODI scores, as well as the intervertebral foramen area measurements, were then used to analyze the effectiveness of the treatment.

### Statistical Analysis

The data were statistically analyzed using SPSS19.0 statistical software. The measurement data were expressed as mean ± standard deviation (X ± SD) and were compared using a paired *t*-test. *P* < 0.05 was considered to be statistically significant.

## Results

A total of 52 cases were included in this study. The age ranged from 48 to 86 years of age, with an average of 76.22 years, while 22 of the patients were male and 30 were female. Among them, seven patients had L3–L4 interspace stenosis, 40 had L4–L5 interspace stenosis, five had L5–S1 interspace stenosis, and six had degenerative spondylolisthesis. For all the patients, the pain was caused by single-space stenosis, with 20 suffering from simple lateral spinal stenosis and 32 from lateral spinal stenosis with intervertebral disc herniation. The patients had been suffering from the disease for between 2 and 50 months, with an average of 8.34 months, while the longest follow-up time was 36 months and the shortest was 12 months, with an average of 19.65 months.

**Figure 6:**
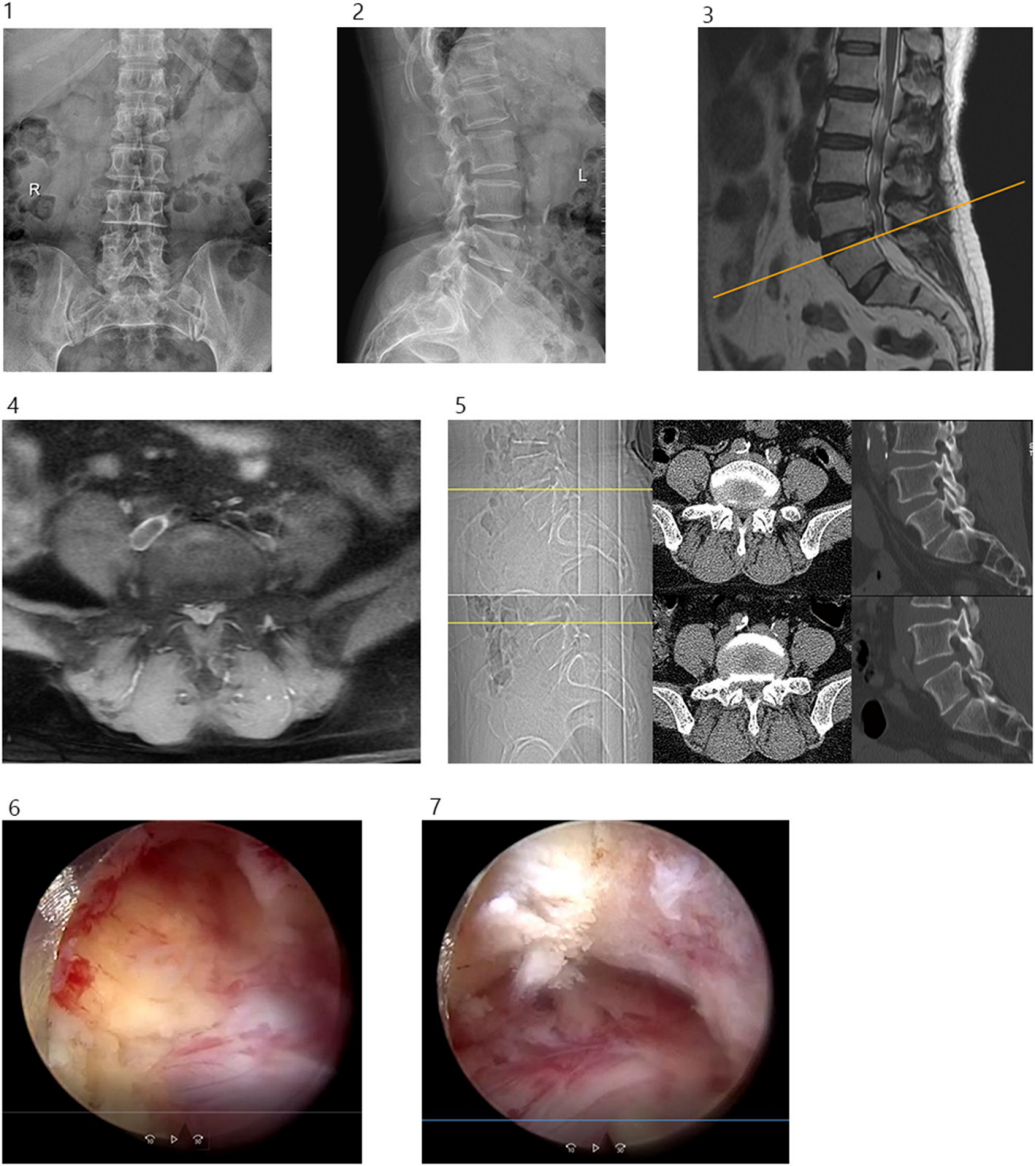
(1) Preoperative lumbar spine anteroposterior projection. (2) Preoperative lumbar spine lateral position. (3 and 4) Preoperative lumbar spine MR showed lumbar 4/5 disc herniation with spinal stenosis. (5) Before and after operation, CT cross-sectional image showed lumbar 4/5 decompression of the right spinal canal, sagittal reconstruction showed partial ventral resection of the superior articular process of L5, partial resection of the upper edge of the vertebral pedicle of L5. (6) Ventral partial resection of the superior articular process of L5 was observed under endoscopy. (7) Partial resection of the upper edge of the pedicle of L5 was observed under endoscopy.

All the patients underwent thin-layer CT scanning prior to the operation and 3 days after. Here, the two-dimensional reconstruction identified that the area of the intervertebral foramen was 422.53 ± 159.20 mm^2^ before the operation and 890.84 ± 367.65 mm^2^ after, which indicated a statistically significant difference (*P* = 0.00*, t* = −9.12). As shown in Table 1, the postoperative ODI was significantly improved at 3 days, 3 months, 6 months, and the final follow-up date (*P* < 0.05), while the VAS scores at all follow-up points after the operation were significantly lower than before the operation (*P* < 0.05). A typical case of simple decompression is shown in Figure 6.

**Table 1:** The VAS score and ODI of 52 patients before and after operation were compared.

	**Preoperative**	**3 days after operation**	**3 months after operation**	**6 months after operation**	**Last follow-up**
VAS	7.80 ± 1.17	2.17 ± 0.57^*^ (*p =* 0.00, *t =* 30.38)	2.10 ± 0.41^*^ (*p =* 0.00, *t =* 32.09)	2.14 ± 0.38^*^ (*p =* 0.00, *t =* 31.72)	1.97 ± 0.36^*^ (*p =* 0.00, *t =* 36.06)
ODI	63.77 ± 9.44	25.12 ± 5.60^*^ (*p =* 0.00, *t =* 35.66)	21.08 ± 3.61^*^ (*p =* 0.00, *t =* 32.36)	20.83 ± 3.96^*^ (*p =* 0.00, *t =* 30.94)	20.27 ± 3.72^*^ (*p =* 0.00, *t =* 30.23)

* *Compared with preoperative, the difference was statistically significant (P < 0.05)*.

## Discussion

The lateral spinal canal is a three-dimensional concept. Here, the lateral boundary is the pedicle of the vertebral arch, the posterior boundary is the superior articular process and the ligamentum flavum, while the anterior boundary is the posterior margin of the vertebral body, the intervertebral disc, and the posterior edge of the lamina terminalis (7). Lee et al. outlined the classification, pathology, and decompression points of lateral spinal stenosis in the so-called open surgery era. The lateral spinal canal is a three-dimensional concept starting from the yellow space of the horizontal disc at the upper edge of the intervertebral disc and extending down to the lateral side of the intervertebral foramen. It can be divided into three segments: entrance-area stenosis, middle-area stenosis, and exit-area stenosis (8), with each having its own anatomical and pathological characteristics.

The first area, the entrance area, extends from the upper margin of the intervertebral disc to the upper margin of the vertebral arch pedicle, with the anterior section of the upper part the intervertebral disc and the posterior part the ventral side of the articular process, with both the internal and external sides open without boundaries. In this part, anterior intervertebral disc degeneration and protrusion, posterior facet hyperplasia, or both, can cause stenosis and induce certain clinical symptoms. The upper part of the entrance area is a movable area and the lower part is an unmovable area, while the anterior part is the posterior edge of the vertebral body and the posterior part is the articular process. The lateral side is open to the intervertebral foramen and the medial side is open to the intraspinal space, while the lower edge lies at the pedicle level. The stenosis of this segment is typically caused by hyperplasia of the articular process or the ligamentum flavum, lip-like hyperplasia of the margins of the vertebral body, or the downward free pulpiform nucleus. Generally, the imaging measurement of lateral spinal stenosis is conducted at the level of the upper edge of the vertebral arch pedicle (6).

Meanwhile, the middle area is the bony channel between the upper and lower edges of the vertebral arch pedicle, with the anterior part of the channel the posterior edge of the vertebral body and the posterior part the lamina, while the lateral side is the vertebral arch pedicle and the medial side is open to the intraspinal space. There is no joint structure in this segment. At the level of the vertebral arch pedicle, the upper edge of the lamina on the medial facet of the articular process acts as the attachment point for the ligamentum flavum. In degenerative diseases, the attachment point of the ligamentum flavum at the upper margin of the lamina is thickened due to accumulated strain and chronic inflammation (9), which results in the dorsal compression of the nerve root of this segment.

Finally, the exit area is the intervertebral foramen area, with the anterior part the posterior edge of the vertebral body, the upper part the lower edge of the pedicle, the posterior part the ventral side of the isthmus of the vertebral arch, and the lower part the upper edge of the lower pedicle of the vertebral arch. In patients with degenerative diseases, the posterior part is the hypertrophic osteophyte of the superior articular process, while the lower border is the hypertrophic osteophyte of the lower lamina terminalis. The stenosis of the exit area is different from that of the entrance area and the middle area, and patients experiencing exit area stenosis were not included in this study.

While the location of the nerve roots from the dural sac varies from segment to segment, the content of the lateral spinal canal below the level of the lower lumbar intervertebral disc entirely consists of nerve roots (10). As such, it is crucial to resect and decompress the hypertrophic and hypertrophic ligamentum flavum in the lower part of the entrance area and the dorsal side of the intermediate region below the intervertebral disc level, which helps to relieve the anterior or posterior compression of the nerve root of the lateral spinal canal and increases the anterior-to-posterior diameter of the lateral spinal canal.

This three-dimensional multiple planar decompression can achieve precise decompression at the entrance area and at the junction between the entrance area and the intermediate area of the lateral vertebral canal, which helps to relieve the compression of the lateral vertebral canal and minimizes the attendant symptoms. When compared to traditional open surgical approach, this procedure has many advantages including reducing muscle injury and postoperative pain, mild surgical trauma, and less operating time (11–14). Intraspinal endoscopic surgery is different from intradiscal surgery since there is no vascular tissue in the intervertebral disc, there is no obvious bleeding in the attendant operation, and the visual field is good. There exist numerous venous plexus on both sides of the anterior vertebral canal. Spinal stenosis often causes the veins to be dilated, and first-dorsal decompression can prevent the blurred vision resulting from vein injury and can reduce any potential nerve damage resulting from radiofrequency hemostasis. Decompression using a circular saw is both highly effective and safe (15) in terms of removing the ventral bone of the superior articular process and decompressing the bony stenosis of the dorsal nerve root.

In this study, all the patients were treated via a posterolateral approach in the lateral position. This approach allows for reaching the lateral vertebral canal directly since no important structures such as blood vessels and nerves are involved (16). For all the patients, local anesthesia was administered, while for the elderly patients, a lateral position local anesthesia was used to improve their surgical tolerance, which proved highly beneficial. In this study, there were 15 patients over the age of 75, which accounted for 29% of the sample (17).

Here, the range of the dorsal decompression of the nerve root was larger than those in previous studies (18) since it extended from the level of the upper margin of the intervertebral disc to the level of the upper margin of the vertebral arch pedicle. This has the advantage of allowing for extensive bony and soft tissue decompression on the dorsal side of the nerve root and is similar to the practice of the authors (19–22). Chronic inflammation and accumulated strain at the attachment point below the ligamentum flavum are major causes of ligamentum flavum thickening (9), and the lack of elasticity in the thickened ligamentum flavum can result in the dynamic stenosis of the vertebral canal (23). Following extensive dorsal bone decompression of the nerve root, the attachment point of the ligamentum flavum at the upper edge of the lamina can be removed and the hypertrophic and hypertrophic ligamentum flavum can be excised, all of which will alleviate the soft tissue compression on the dorsal side of the nerve root.

An accurate diagnosis and adequate decompression are two important factors in percutaneous endoscopic spine surgery. Elderly patients often suffer from multiple segmental degeneration, and SNRB was used to identify the responsible nerve roots in the patients suffering from this degeneration in this study before endoscopic decompression was performed in the second stage. These measures improved the accuracy and, provided an adequate decompression is performed following the diagnosis, this can ensure the best postoperative effect.

With the popularization of thin-layer CT and the development of the applicable software, CT sagittal plane reconstruction and area measurement have become considerably more convenient. In this study, the posterolateral approach was used to decompress the dorsal bone structure of the nerve root in the entrance area and at the upper edge of the intermediate area. Here, the structure is different from that of the dura mater and the intervertebral foramen, and it can prove extremely difficult to quantitatively measure this area without marker points, with the measurement error of different scholars consistently large (24). Given that the structure of the articular process of the lateral vertebral canal itself presents the extension of the superior articular process to the medial side, and since the structure of the upper and lower pedicles of the vertebral arch is constant, the area measurement of the medial margin of the intervertebral foramen is relatively stable. In view of this, in this study, the area of the intervertebral foramen between the upper and lower pedicle of the vertebral arch and the medial margin of the pedicle was used to replace the sagittal area of the lateral spinal canal. The images were reconstructed via a CT soft-tissue window before and after the operation and the area of intervertebral foramen was measured using sagittal reconstruction. There were significant increases after the operation in with the differences found to be statistically significant (*P* < 0.05).

In addition, there is a certain correlation between the improvement of clinical symptoms and the area of intervertebral foramen (25). Bone hyperplasia at the posterolateral edge of vertebral body and facet joint hyperplasia can cause intervertebral foramen stenosis, oppress nerve roots, and cause clinical symptoms. The imaging manifestations are the decrease of sagittal area and anteroposterior diameter of intervertebral foramen, which is consistent with the results reported by Wildermuth et al. (26).

## Conclusions

In summary, transforaminal approach multiple planar endoscopic decompression can achieve an accurate and effective decompression of the lumbar lateral spinal canal, has good short-term effects, and is especially suitable for elderly patients. There are some limitations in our study. The small sample size and short follow-up may limit the comparability and outcomes. What's more, we have not differentiated patients with pure bony stenosis or mixed stenosis (bony+disc) and performed sensitivity analysis. Therefore, future studies with longer follow-up and larger simple size are needed.

## Data Availability Statement

The raw data supporting the conclusions of this article will be made available by the authors, without undue reservation.

## Ethics Statement

This study was conducted with approval from the Ethics Committee of the Second Affiliated Hospital of Jiaxing University. The patients/participants provided their written informed consent to participate in this study.

## Author Contributions

H-gL, X-kP, and M-jH conceived the idea and conceptualized the study. J-qZ, J-mS, and BC collected the data. XZ and YY analyzed the data. H-gL and X-qH drafted and reviewed the manuscript. All authors read and approved the final draft.

## Conflict of Interest

The authors declare that the research was conducted in the absence of any commercial or financial relationships that could be construed as a potential conflict of interest.

## Publisher's Note

All claims expressed in this article are solely those of the authors and do not necessarily represent those of their affiliated organizations, or those of the publisher, the editors and the reviewers. Any product that may be evaluated in this article, or claim that may be made by its manufacturer, is not guaranteed or endorsed by the publisher.
